# Fast Prototyping Microfluidics: Integrating Droplet Digital Lamp for Absolute Quantification of Cancer Biomarkers

**DOI:** 10.3390/s20061624

**Published:** 2020-03-14

**Authors:** Beatriz Oliveira, Bruno Veigas, Alexandra R. Fernandes, Hugo Águas, Rodrigo Martins, Elvira Fortunato, Pedro Viana Baptista

**Affiliations:** 1 UCIBIO, Departamento de Ciências da Vida, Faculdade de Ciências e Tecnologia, Universidade NOVA de Lisboa, Campus de Caparica, 2829-516 Caparica, Portugal; abb.oliveira@campus.fct.unl.pt (B.O.); bmrveigas@gmail.com (B.V.); ma.fernandes@fct.unl.pt (A.R.F.); 2 i3N|CENIMAT, Departamento de Ciência dos Materiais, Faculdade de Ciências e Tecnologia, Universidade NOVA de Lisboa, Campus de Caparica, 2829-516 Caparica, Portugal; hma@fct.unl.pt (H.Á.); rfpm@fct.unl.pt (R.M.); emf@fct.unl.pt (E.F.)

**Keywords:** digital amplification, integrated microfluidics device, loop-mediated isothermal amplification, oncogenes, lab-on-a-chip

## Abstract

Microfluidic (MF) advancements have been leveraged toward the development of state-of-the-art platforms for molecular diagnostics, where isothermal amplification schemes allow for further simplification of DNA detection and quantification protocols. The MF integration with loop-mediated isothermal amplification (LAMP) is today the focus of a new generation of chip-based devices for molecular detection, aiming at fast and automated nucleic acid analysis. Here, we combined MF with droplet digital LAMP (ddLAMP) on an all-in-one device that allows for droplet generation, target amplification, and absolute quantification. This multilayer 3D chip was developed in less than 30 minutes by using a low-cost and extremely adaptable production process that exploits direct laser writing technology in “Shrinky-dinks” polystyrene sheets. ddLAMP and target quantification were performed directly on-chip, showing a high correlation between target concentration and positive droplet score. We validated this integrated chip via the amplification of targets ranging from five to 500,000 copies/reaction. Furthermore, on-chip amplification was performed in a 10 µL volume, attaining a limit of detection of five copies/µL under 60 min. This technology was applied to quantify a cancer biomarker, *c-MYC*, but it can be further extended to any other disease biomarker.

## 1. Introduction

The increasing demand for faster and inexpensive diagnostic tools toward integrated devices has been pushed forward by combining microfluidics with molecular detection approaches, both considered key features of lab-on-a-chip (LOC) technology [1]. These integrated LOC platforms bring numerous advantages, mostly due to the scale effect such as enhanced sensitivity, throughput, and portability, while reducing the costs and sample volume [1].

Nucleic acid amplification technologies (NAATs) benefit from microfluidics integration, which allows for faster and decentralized analysis of molecular disease biomarkers, especially those requiring the detection and quantification of DNA/RNA targets. From these, digital amplification systems are today changing the field of molecular sensing, particularly those that rely on dividing a solution containing the nucleic acid molecules and the reaction components into numerous discrete reaction vessels: droplets [2,3]. Considering that the number of target molecules inside each droplet follows a Poisson distribution, the absolute quantification of initial target molecules is estimated after endpoint amplification by modeling the fraction of positive droplets [4]. 

So far, polymerase chain reaction (PCR) has been the most widely exploited nucleic acid amplification system, be it on its conventional, quantitative, or digital arrangements [5]. Nevertheless, PCR requires thermal cycling with temperatures as high as 95 °C, resulting in complex and cumbersome challenges in the design and operation of chip-based systems. Conversely, isothermal amplification schemes remove the need for temperature control instrumentation, while reducing the amplification time, thus making them more suitable for screening scenarios [4]. From these, LAMP is the most commonly used method as it provides an outstanding specificity when compared with other isothermal amplification architectures due to the requirement of four to six primers [6]. The potential of LAMP-on-a-chip relies on the simplicity of its thermal requirements, easy integration with sample preparation steps, multiple detection methods and tolerance to biological components present in clinical samples. Although paper-based and digital variants of LAMP-on-a-chip have been proposed [7,8], the feasibility of false positive phenomena is a shortcoming concerning the LAMP mechanism, which is outmatched by adding an extra step of sequence recognition, often accomplished with specific fluorescence probes or by restriction enzyme profiling [9]. 

Chip-based microfluidic systems have played an important role in droplet generation technology for biological, biomedical, and diagnostic applications [10,11,12,13]. Droplet-based microfluidics uses microchannels and immiscible fluids to generate discrete fractions of aqueous solutions. One of the most widely used geometries is the flow-focusing [10,14], in which the dispersed phase is symmetrically sheared by the continuous phase in the cross-junction [10,15]. This architecture yields highly monodisperse droplets [16,17] that can be used in a diverse range of applications [12]. Devices exploiting this microchannel geometry are also commercially available (e.g., Bio-Rad QX200, Stilla Naica, Raindance Raindrop Plus). Nevertheless, these devices are costly and, for screening more than one target at a time, require multiple apparatus that are not easily integrable. Additionally, the requirement for specific proprietary materials and reagents have been limiting the adoption of this technology into a larger scale.

Microfluidic chip fabrication and optimization have not been easily accessible to everyone. Standard production processes have been based in lithographic techniques [18,19,20]. These methods pose several limitations in a R&D environment, since they mostly rely on cumbersome, lengthy, and expensive fabrication protocols; additionally, they also present limited design flexibility [21]. Recently, several methods have been developed toward fast prototyping of multilayer microfluidic chips [22,23,24,25]. From these, direct patterning of complex three-dimensional, stacked polystyrene (PS) microfluidic chips has been accomplished [26]. By leveraging the inherent shrinkage properties of biaxially pre-stressed thermoplastic sheets, microfluidic channels become thinner and deeper upon heating [26]. These advantages make PS a very promising material to fabricate microfluidic devices, although there is a need for inexpensive and fast processes that allow for polystyrene micro-structure fabrication [25]. On this, laser direct-writing micromachining has become a promising alternative to lithography, because of its low cost, fast speed, scalability, and non-contact characteristics [27,28]. CO_2_ laser systems have been widely used for rapid production of microfluidic systems with several different polymers (e.g., PS, poly(methyl methacrylate) (PMMA), polydimethylsiloxane (PDMS), polycarbonate (PC), and polytetrafluoroethylene (PTFE)) [29,30,31,32,33]. While several of these fabrication concepts have already been reported, no research has been pursued toward their application in integrated molecular detection approaches. 

Despite all the recent breakthroughs in the molecular mechanisms of cancer, there is still a pressing demand to develop platforms, especially for earlier cancer diagnosis, that combine the specificity and accuracy of molecular methods with portability, user friendly, and cost-effective features such as those presented by LoC technologies [34]. Cancer is one of the leading causes of death in the developed world, as estimated by the World Health Organization (WHO) [35]. This disease is generally defined as a consequence of multiple genetic events that can exert two broad effects: gain of function mutations leading to oncogenes (e.g., *c-MYC*, *KRAS*, HER2, and *SRC*) [36] and loss of function mutations resulting in the inactivation of tumor-suppressor genes (e.g., *Tp53*, *RB1*, and *CDKN2A*) [37]. Constitutive expression of the proto-oncogene *c-MYC* plays an important role in tumor progression and has been associated with a variety of hematopoietic tumors, leukemias, and lymphomas including Burkitt lymphoma [38]. The protein encoded by this gene controls the regulation of the cell cycle and cell growth, activating genomic instability, stimulating angiogenesis, cell transformation, and apoptosis [38]. As proof-of-concept, the *c-MYC* proto-oncogene was used in this research. 

Herein, we demonstrate a novel approach for multilayered PS-based microfluidic device production that integrates an inexpensive and fast fabrication process based on the shrinkage properties of PS. This integration significantly increases the design flexibility while reducing the prototyping time and cost of the device. As such, we demonstrate the use of this approach for a fully functional droplet-based microfluidic chip and its application with the aim of an integrated ddLAMP assay for absolute target quantification. Additionally, the innovative incorporation of multilayered channels with fully transparent chambers allows for in situ detection of the amplification. The principle here demonstrated can also be applied to absolutely quantify other disease biomarkers and further gene expression analysis. 

## 2. Materials and Methods

### 2.1. Material and Reagents 

The droplet generation oil for Evagreen^®^ was purchased from Bio-Rad Laboratories, Inc., (Hercules, CA, USA). Evagreen dye was obtained from Biotium Inc. (Fremont, CA, USA). dNTPs were purchased from Bioline (London, UK). Betaine and magnesium chloride were acquired from Sigma-Aldrich (St. Louis, MO, USA). The DNA size marker GeneRuler^TM^ DNA Ladder Mix was purchased from Fermentas (Burlington, ON, Canada). Polystyrene thermoplastic sheets “Shrinky-Dinks” were obtained from K&B Innovations (North Lake, WI, USA). All primers were purchased from STAB VIDA, Lda., (Caparica, Portugal).

### 2.2. Sample Source and Preparation of Template DNA

Genomic DNA (gDNA) from the HCT-116 cell line was extracted following the phenol-chloroform method [39]. A 229-base pair (bp) fragment of the human *c-MYC* proto-oncogene (Ac. No. NM_002467) associated to cancer development [40] was amplified from the extracted gDNA by PCR using the MYC-forward (5’-GCTCATTTCTGAAGAGGACTTGT-3’) and MYC-reverse (5’-GGCAGTTTACATTATGGCTAAATC-3’) primers. PCR amplification was performed on a Bio-Rad MyCycler Thermocycler in a 20 μL final volume using 1 μM of the specific primers, 2.5 mM dNTPs with 1 U Taq DNA Polymerase (GE Healthcare Europe, Germany), with the following thermal cycling conditions: initial 5 min denaturation at 95 °C, followed by 24 amplification cycles of denaturation at 95 °C for 30 s, annealing at 62 °C for 30 s, elongation at 72 °C for 30 s, and a final elongation at 72 °C for 5 min [41]. Following purification of the amplified fragment by ethanol precipitation [42], an agarose gel electrophoresis stained with GelRed 1X was performed and the concentration (in copies per µL) was determined by pixel intensity/counting using ImageJ imaging software (https://imagej.nih.gov/ij/) (NIH, Bathesda, MD, USA). Determination of the copy number of amplifications was performed by linear regression analysis by fitting the fluorescence intensity of the amplicon band to a calibration curve based on the fluorescence intensity of the ladder bands (see Figure A1).

### 2.3. Benchtop LAMP Amplification of c-MYC

The *c-MYC* fragment previously PCR-amplified was used as a template for the LAMP reaction, performed as described by Veigas et al. [41]. This LAMP reaction requires four specific primers: a forward outer primer (FP), a backward outer primer (BP), a forward inner primer (FIP), and a backward inner primer (BIP) (see Table B1 for primers). LAMP primers for *c-MYC* were designed using Primer Explorer V4 (http://primerexplorer.jp/elamp4.0.0/). The reaction was carried out in a 10 µL reaction mixture containing 1.6 µM of FIP and BIP primers, 0.2 µM of B3 and F3 primers, 0.45 mM of dNTPs, 3 mM of MgCl_2_, 0.8 M of Betaine, 700 µM of dNTPs, 1 X Bst Buffer, 1 X Evagreen dye, 1.8 U of Bst polymerase (New England Biolabs, Beverly, MA), and 1 µL (5–500,000 copies/µL) of template DNA; sterile water was used in place of DNA for the non-template control (NTC). The reaction was incubated at 65 °C for 60 min in a Bio-Rad MyCycler Thermocycler.

### 2.4. Chip Design and Fabrication Process

**Figure 1:**
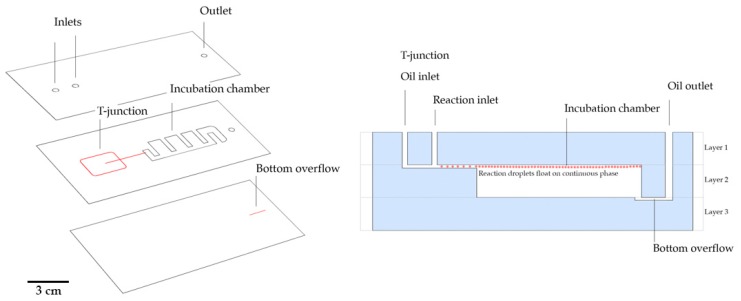
Polystyrene (PS) multilayer microfluidic chip fabrication. Each layer was engraved (using a CO_2_ laser machine). The layers were then aligned and thermally bonded and further placed in the oven for simultaneous shrinking. (**Left**) Layer design and features. Black: cut specification – laser power = 35%; Red: channel engraving – laser power = 8%. Scale bar represents 3 cm. (**Right**) Side view cut of the assembled chip.

**Figure 2:**
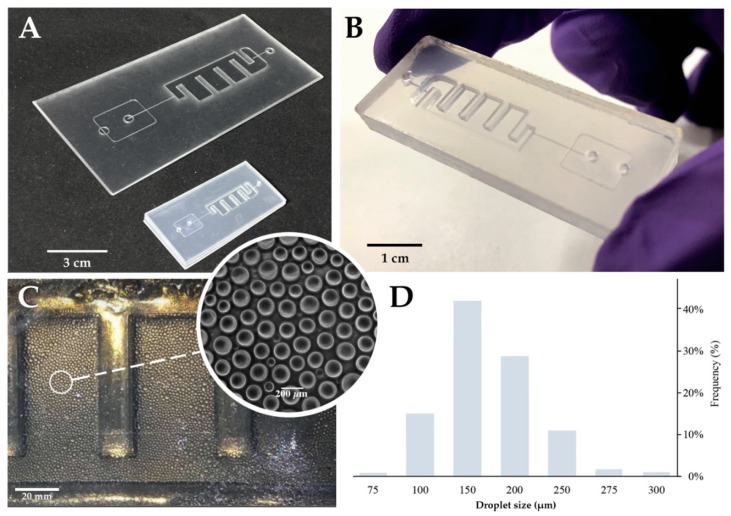
Multilayer Chip production and performance. (**A**) Multilayer chip before and after the shrinking process; the shrink was isotopically in plane and reduced around 66% of the original size. Additionally, this occurred in an increase in the height over 500%. Scale bar represents 3 cm. (**B**) Final chip appearance after frame removal. This step also helps seal the edges. Scale bar represents 1 cm. (**C**) On-chip droplets. Scale bar represents 20 mm; (inset) bright-field microscope image of the produced droplets inside the incubation chamber Scale bar represents 200 µm. (**D**) Frequency distribution of the droplet sizes. Droplets present a weighted average size of 170 μm, a coefficient of variation equal to 3% and a standard error of mean (SEM) of 0.27. Plot of the droplet size distribution for all the experiments (n = 6000 droplets). The mean volume of each droplets is ~2.6 nL.

Chips were initially designed on a vector image software (Adobe illustrator, Adobe systems software, Ireland) and channels were engraved on transparent PS sheets on a computer controlled–CO_2_ laser machine (VLS 3.50, Universal Laser Systems, Vienna, Austria), with a 10.6 μm wavelength and a beam diameter of 0.127 mm, 50 W of power, and 0.254 m/s writing speed at 1000 ppi (pulses per inch). As shown in Figure 1, the device is composed of three layers with two laser cutting specifications: Black: cut specification—laser power = 35%; Red: channel engraving—laser power = 8% with a parallel line design with a 400 µm spacing (final channel width of ~200 µm after shrinkage); for channel engraving characterization see Figure C1 and Figure 2. The top layer has two inlets and one outlet. The middle layer has a microchannel engraved with flow-focusing geometry connected to an incubation chamber with the full width of the PS sheet. Channels were produced with a width and height of 200 μm, thus, an aspect ratio of 1 (h/w = 1). The incubation chamber in the middle layer had a total capacity of 300 μL. The bottom layer consisted of a flat surface to seal the incubation chamber and a bottom channel for oil overflow, which is connected to the outlet present in the two previous layers. After patterning, each layer was aligned and placed between two slabs of Teflon. The assembled unshrunken layered chip was thermally bonded on a hot press for 20 minutes at 110 °C. After 20 minutes, the chips were removed from the hot press and placed in an oven at 155 °C on a Teflon plate for five minutes. After shrinkage, the chips were allowed to cool down at room temperature for two minutes. An extra step of laser cutting was performed to remove the excess material around the chip frame and create an extra seal between layers (one minute). 

### 2.5. Droplet Generation and Chip Operation Conditions

Two syringe pumps were used to infuse two different solutions (continuous and dispersed phase) (Legato210P, KDScientific, Holliston, MA, USA). The continuous phase flow rate (*Qc*) and dispersed phase flow rate (*Qd*) were set at *Qc* = 20 µL/min and *Qd* = 1 µL/min. Small changes to the initially *Qd*/*Qc* ratio were made to modulate droplet size [10]. The syringe pumps were connected to the chips’ inlets via microfluidic tubes (LVF-3480, Darwin microfluidics, Paris, France). Chip operation started by filling the chip with the continuous phase *Qc* = 20 µL/min (QX200^TM^ Droplet generation oil for Evagreen, BioRad, Hercules, CA, USA), after which the second syringe started with a flow of *Qd* = 1 µL/min for a flow ratio of 1:20. The second syringe was previously filled with 10 µL of LAMP reaction with 1X Evagreen dye. A draining tube was connected to the outlet for oil overflow. After sample infusion, tubes were detached from the inlets and outlet, and then sealed with hot glue.

### 2.6. On-Chip LAMP Reaction Integration

One of the key aspects of ddLAMP technology is droplet production with reliable control over size. ddLAMP usually resorts to portioning the reaction volume into 20,000 nanoliter-sized droplets with extremely low size dispersion. For this, a flow of *Qc* = 20 µL/min and *Qd* = 1 µL/min was used for the oil and sample inlet, respectively. LAMP reaction was performed by adding 10 µL of the reaction volume for DNA concentrations ranging from five to 500,000 copies/µL. After droplet generation, the chips were sealed with hot glue and the reaction temperature was kept at 65 °C (bottom plate heating) for 60 minutes. Endpoint amplification was measured with a minimum of four independent areas of the chip in a fluorescence microscope (Nikon Eclipse Ti-U inverted microscope, Nikon, Japan).

### 2.7. Data Analysis

After microscope image acquisition, data were analyzed in ImageJ software by measuring the area and grey mean value (GMV) parameters. For each target concentration, a minimum of two independent experiments and 400 droplets per experiment were considered. The area measurements were converted to diameter and the GMV of each droplet was normalized by dividing it by the average of three GMV measurements of the image background. Next, the normalized GMVs were plotted as a function of the corresponding droplet´s diameter. The threshold line with the equation of y = 0.001x + 1.14 was used to score positive droplets (droplets above the threshold) and negative droplets (droplets below the threshold). The statistical analysis of the positive droplets implies the correction of the scored fraction of positive droplets to the Poisson probability of a droplet containing zero targets, following the equation:

*E^C^_pos_* (%) = 1- (*N_neg_* ∗ Pr (0)/N) ∗ 100 (1)

where *E^C^_pos_* gives the fraction of positive droplets corrected to Poisson’s statistics; *N_neg_* is the number of negative droplets; N the total number of droplets; and Pr(0) is the Poisson’s probability of a droplet containing zero target molecules for each target concentration (see Figure D1).

## 3. Results and Discussion

### 3.1. Chip Design and Fabrication Process

Most of the microfluidic devices require complicated fabrication processes such as photolithography, hot embossing, and injection molding. In R&D settings, there is a continuous need to change/optimize chip design, so the improvement of prototyping time and costs is of much value. This microfluidic chip was produced with a laser ablation mechanism in shrinkable PS sheets, followed by thermal bonding to assemble the 3D multilayered chip [43]. The reported approach allows for fast (under 30 min) production of functional chips, while eliminating cumbersome fabrication steps. Additionally, it also enables the creation of integrated structures with high design flexibility, thus lowering material expenditure and reducing the costs of chip development (under 50 cents per chip for the proposed design). Even though the fabrication process here describes the results from combining two previously reported techniques: CO_2_ laser engraving [29] and “Shrinky-Dinks” PS sheets [25], what is truly compelling is the advantage given by the multilayered design, which can not only be adapted for several applications, but also enables the creation of fully transparent chambers suitable for on-chip and real-time optical data acquisition. Furthermore, this is the first time that these techniques have been translated into a functional biodetection platform.

We used shrinkable PS sheets because it allowed us to design the channels in a “large” scale version that were then heat-shrunk (~66% of size reduction) (Figure 2A), contributing to an increase in the X and Y resolution while achieving precise control over the channel height. The 3D chips were designed with the aim of an all-in-one integrated platform (Figure 2B) for droplet generation, amplification reaction, and detection. For this, channels on both the top and bottom sections, and a full depth incubation chamber were engraved (Figure 2C). The incubation chamber is enclosed (top and bottom) by a single sheet of PS, increasing the transparency, and reducing the optical defects that could interfere with fluorescence acquisition. Additionally, the bottom overflow channel allowed for a continuous operation without the loss of droplets (droplets float in the oil phase).

The profile of the microchannel depends on the intensity distribution of the laser beam given by laser power, cutting speed, and number of beam paths on the same channel. Single line designs show a cross section with an extremely sharp Gaussian distribution. To circumvent this effect, a parallel vector design was used and optimized for a 1:1 depth/width channel ratio design (see Figures C1 and C2). The engraved channels present a 200 μm width and height, that are suitable to generate droplets of 150-200 μm in diameter (Figure 2D). Flow rates were optimized to generate droplets with an average size of approximately 170 μm in diameter (corresponding to ~2.6 nL). This size range correlated to those obtained with the standard commercially available devices for digital droplet PCR (dd PCR) [44]. Additionally, the droplets showed a narrow polydispersity and a low coefficient of variation (CV = 3%; defined as CV = standard deviation/mean), which were among the lowest polydispersity obtained with microchannel droplet generation schemes (usually 1–3%) [45].

### 3.2. Chip-Based Droplet Digital Lamp

Following the design and fabrication of the device, this platform was applied to a digital LAMP approach. Digital amplification techniques are based on the partitioning of the amplification reaction into many small reaction vessels, where amplification occurs. The absolute quantification of the initial sample concentration was achieved through a Poisson statistical analysis of the positive droplets (droplets holding at least one target molecule) versus negative droplets (droplets with zero target molecules) [46], which requires the proper distinction of these two populations.

**Figure 3:**
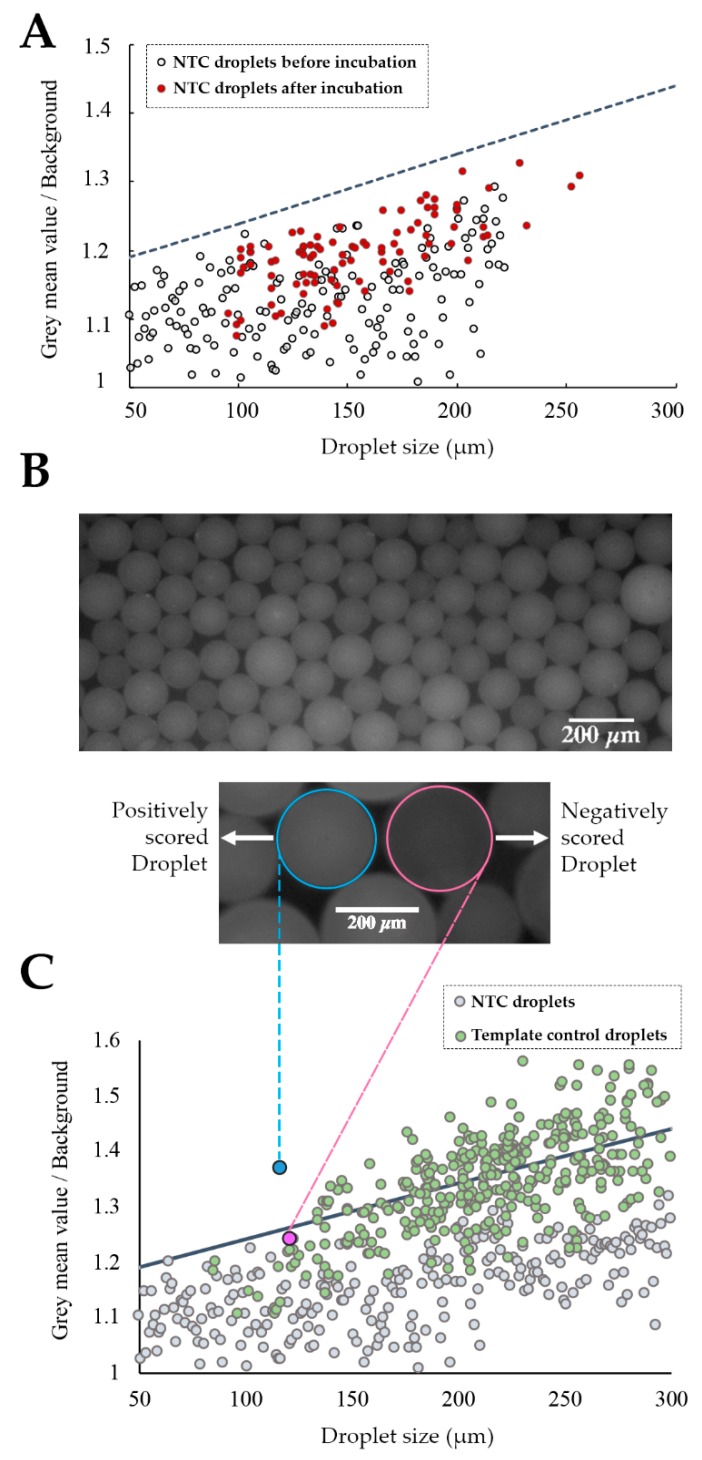
Chip-based ddLAMP. (**A**) Threshold definition; fluorescence measurements of droplets without a template before and after end-point reaction. (**○**) Represents the NTC droplets before incubation at 65 °C and (●) Represents the NTC droplets after incubation at 65 °C. The threshold (**---**) equation is y = 0.001x + 1.14. (**B**, **top**) Fluorescence image of on-chip ddLAMP droplets from a template sample after end-point amplification. (**Bottom**) Representative fluorescence image of a (○) negative droplet vs. a (○) positive droplet. Scale bar of 200 μm in length; (**C**) Assessment of the threshold differentiation capability. Negative/positive droplet score. Only droplets with fluorescence above the threshold were scored as positive. (●) Represents droplets from the NTC sample after end-point reaction. (●) Represents droplets from the template sample after end-point LAMP reaction. (●) Represents a negatively scored droplet from the template sample (fluorescence image shown in Figure 3B and (●) represents a positively scored from the template sample (fluorescence image shown in Figure 3B).

For this, a threshold line was set to allow scoring the positive from negative droplets based on their fluorescence amplitude. Positive droplets inherently exhibit higher fluorescence than negative droplets due to the presence of Evagreen, a dsDNA binding dye in the amplification reaction. However, the fluorescence amplitude of Evagreen fluctuates depending on the droplet size, amplicon size, amplification efficiency, and primer-dimer formation [47]. Furthermore, larger droplets show an increased probability of encompassing more Evagreen molecules, yielding higher basal fluorescence [44]. Therefore, to define the threshold for droplet scoring, several reactions without template molecules were assessed (Figure 3A). Data showed a clear correlation between the fluorescence and droplet size, indicating that the threshold line must have a two-dimensional equation with a positive slope value [48].

Figure 3A demonstrates that droplets from the NTC reaction did not present a significantly increased basal fluorescence at end-point reaction. Thus, the threshold line was iteratively defined taking into account all the attained baseline measurements to distinctively distinguish positive from negative droplets. To confirm if the defined threshold was suitable to discern between negative and positive droplets (Figure 3B), this was applied to the output of both (NTC and template) reactions (Figure 3C). These results show that the defined threshold allows for the differentiation of positive from negative droplets, since only droplets from the template control sample have fluorescence values above the threshold, thus being scored as positive while all of the droplets from the NTC have a fluorescence below the threshold (negatively scored droplets). Nevertheless, some droplets from template control also present fluorescence values below the threshold, which may be attributed to the Poisson distribution of target molecules (i.e., some of these droplets do not hold any target molecules, while some of the positive droplets may contain more than one).

### 3.3. Device Application on Target Quantification

**Figure 4:**
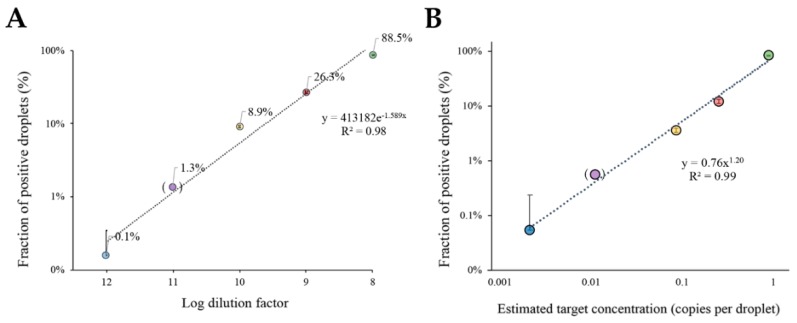
Target DNA quantification with ddLAMP. (**A**) Fraction of positive events attained with Poisson-based correction for different target dilutions. The fraction of positive events corrected to the Poisson statistics are represented in a logarithmic scale. The trendline presents an exponential equation: y = 413182e^−1.589X^ and a R^2^ = 0.98. Data were obtained through the measurement of the area of the droplets and corresponding mean grey value with ImageJ software. (**B**) The plot of the positive droplet fraction against the expected DNA concentration in copies per droplet shows an exponential relationship predicted by Poisson distribution. The trendline for the power adjustment has a R^2^= 0.99 and an equation: y = 0.76x^1.20^. Copy number for each dilution was calculated with the equation *C = −ln* (1 *– E_pos_*). For both panels: The error bars correspond to the standard deviation of two independent experiments (n = 2) with 400 measured droplets for each reaction; dilution factor 11 (between brackets) was only performed once (n = 1). (●) Represents the 10^−12^ dilution, (●) Represents the 10^−11^ dilution (●) Represents the 10^−10^ dilution, (●) Represents the 10^−9^ dilution, and (●) Represents the 10^−8^ dilution.

To evaluate the effectiveness of this chip on a ddLAMP approach for target quantification, serial dilutions of a selected target DNA were analyzed (logarithm of dilution factor ranging from −6 to −12). As a model, *c-MYC* oncogene was used, since this is a valuable biomarker of malignant transformation leading to cancer development [38], however, any other genetic marker could be applied. End-point amplification reactions were conducted for the above-mentioned template dilutions, followed by fluorescence imaging and data processing. Figure 4A shows the increase of the fraction of positive droplets (*E_po_*_s_) as a function of the sample dilution factor. These results were obtained by modeling the fraction of positive droplets to Poisson partitioning statistics (see Figure D1). The increase of *E_pos_* occurs due to a rise in the number of target molecules, and consequently, the probability of a droplet containing at least one target molecule also increases, which in turn leads to an increase in the number of fluorescence droplets after the end-point reaction. The algorithm for scoring positive droplets correlates perfectly to the defined threshold, yielding a very robust correlation to target dilution (R^2^ = 0.98). This indicates that the designed chip is capable of discriminating the target concentrations for a dynamic range of five orders of magnitude. Nevertheless, the attained dynamic range is only constrained by the number of droplets, which can be easily adjusted by scaling the incubation chamber.

A similar approach was then used for absolute quantification of *c-MYC* molecules in each sample. The percentage of positive droplets as a function of estimated target DNA concentration provides a robust calibration curve (Figure 4B). The plot shows a high linear correlation factor between the fraction of positive droplets and the predicted number of molecules (per droplet) given by the Poisson distribution. As a result, not only is the chip design suitable for direct ddLAMP, but the developed algorithm also provides a simple and straightforward quantification of the target molecules in the samples. It should be noted that the plot in Figure 4A,B only shows the target dilutions for five orders of magnitude. Taking this into consideration, for each additional order of magnitude, the number of droplets should be increased 10-fold.

Using this chip approach, it was possible to determine the concentration of template target DNA by assessing only a total of 400 droplets. This is an improvement compared to the commercially available devices that require the measurement of thousands of droplets/events for the same dynamic range [49]. As such, the device herein proposed is capable of delivering similar results, but without the need for complex and expensive detection units. In fact, our approach was capable of quantifying the target concentration down to 0.001 copies/droplet corresponding to 5 copies/μL.

## 4. Conclusions

Herein, an integrated chip for digital nucleic acid detection system relying on isothermal LAMP reaction in a digital readout strategy was presented. The fabrication strategy exploits two previously reported techniques. Their combination allows for fast chip development and testing, which is particularly beneficial in R&D settings. In fact, one of the major advantages of this approach is the fast-interactive development of multilayered chips under 30 minutes. Moreover, this is the first time that this production scheme is applied into a working biodetection platform. The proposed design allows for uniform droplet generation, amplification, and measurement of the resulting fluorescence in a single device. This innovative chip-based ddLAMP exhibited the capability of distinguishing between different target concentrations, paving the way for comparative determinations of target DNA/RNA such as those relating to gene expression analysis, which is an essential feature for the quantification of nucleic acid biomarkers in cancer diagnostics.

The laser ablation technique, together with the use of biocompatible shrink PS sheets, allowed for the production of a flow focusing droplet generator, which attained droplets with a coefficient of variation of 3% and average size of ~170 µm, corresponding to a volume of ~2.6 nL. The included fully transparent chamber allows for clear fluorescence image acquisition, contributing to correct droplet score. The current setting allows for the incubation of roughly 6000 droplets, providing for target quantification in a working range of five decades. Nevertheless, this range is only limited by the number of droplets produced, which in turn is only restricted by the size of the incubation chamber. Further optimizations (chamber size and design) are extremely easy to implement and fabricate, making this an attractive alternative to the traditional fabrication processes for the R&D scenario.

Still, some aspects should be improved in order to enhance the score assessment such as the optimization of droplet generation to remove size dispersity, optimization of the LAMP reaction conditions (i.e., primer concentration), lowering the basal fluorescence of the droplets, and using an external reference dye to normalize the initial fluorescence of the droplets. Moreover, the current setting requires a fluorescence microscope for image acquisition. Nonetheless, in the future, we envision its integration with commercially available portable measurement systems, aiming at a truly standalone biodetection platform. This approach can be further advanced into a multiplex platform, allowing for seamless processing of multiple samples on a single device, thus achieving gene expression analysis capability. Despite this 3-layered design, previous tests showed the capability of fabricating functional devices with up to five layers, increasing the design freedom for any forceable application.

## Supplementary Materials

The following are available online at https://www.mdpi.com/1424-8220/20/6/1624/s1, Figure A1: Copy number estimate based on gel electrophoreses, Table B1: List of LAMP primers, Figure C1: Effect of laser power in microfluidic channel optimization, Figure C2: Effect of single vs. double line in microfluidic channel optimization, Figure D1: Poisson partitioning statistics.
