# Supplementary material for: Fast Prototyping Microfluidics: Integrating Droplet Digital Lamp for Absolute Quantification of Cancer Biomarkers
Source: Sensors (Basel). 2020 Mar 14;20(6):1624. doi: 10.3390/s20061624 (PMC7146133; doi:10.3390/s20061624)
Supplement: Supplementary file 1 [file sensors-20-01624-s001.pdf]

Supplementary Materials

Fast Prototyping Microfluidics: Integrating Droplet Digital Lamp for Absolute Quantification of Cancer Biomarkers

Beatriz B Oliveira, Bruno Veigas, Alexandra R. Fernandes, Hugo Águas, Rodrigo Martins, Elvira Fortunato and Pedro Viana Baptista

Appendix A: Target Copy Number Quantification

Copy number was calculated based on band density relating to the quantification of the original target sample after agarose gel electrophoresis.

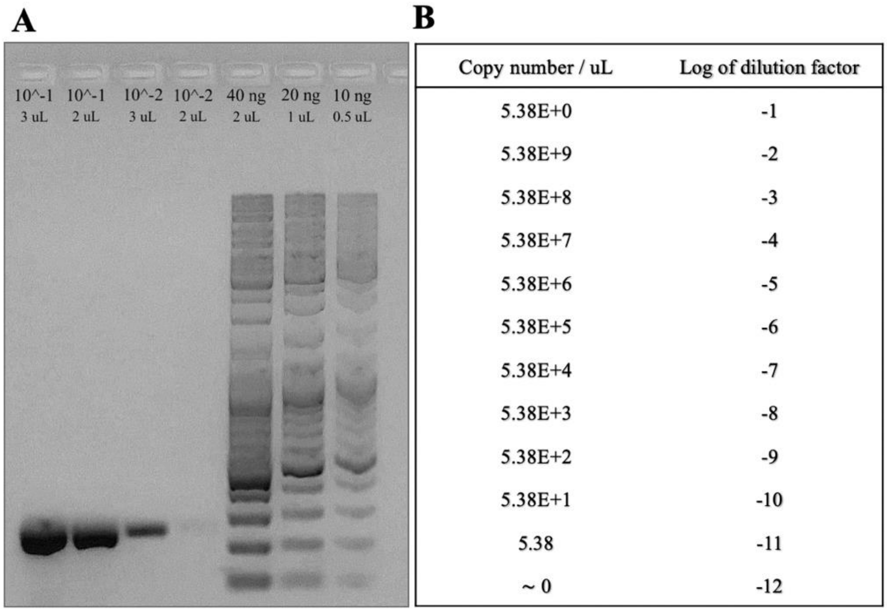

Copy number / uL	Log of dilution factor
5.38E+0	-1
5.38E+9	-2
5.38E+8	-3
5.38E+7	-4
5.38E+6	-5
5.38E+5	-6
5.38E+4	-7
5.38E+3	-8
5.38E+2	-9
5.38E+1	-10
5.38	-11
~ 0	-12

Figure A1. Copy number estimate based on gel electrophoreses. (**A**) Gel electrophoreses of the c-Myc PCR product with −1 and −2 logarithm of dilution factors, used for copy number calculations. (**B**) Copy number of c-Myc PCR products per μL with several serial dilutions.

Appendix B: LAMP Primer Set

Table B1. List of the LAMP primers.

Name	Sequence
c-MYC F3	5′-TCTGAAGAGGACTTGTTGC-3′
c-MYC B3	5′-TTCAGTCTCAAGACTCAGC-3′
c-MYC FIP	5′-CTTTTCCTTACGCACAAGAGTTCC-GGAAACGACGAGAACAG-3′
c-MYC BIP	5′-ACGATTCCTTCTAACAGAAATGTCC- CAAGGTTGTGAGGTTGCA-3′

Appendix C: Microfluidic Channel Optimization

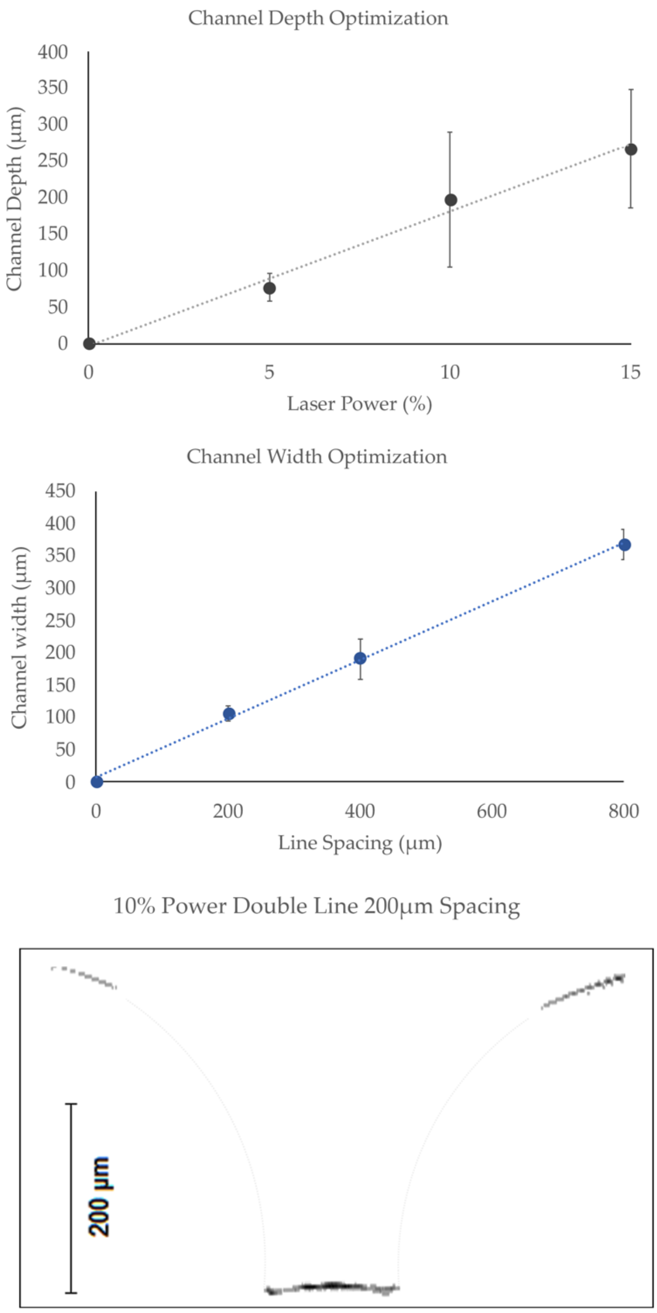

Figure C1. Effect of laser power in microfluidic channel optimization. Effect of laser power on channel depth and the effect of line spacing (double line design) on channel width. (**Bottom**) Confocal microscope z-stack image of a channel produced with 10% power and 200 μm line spacing (channel width at the bottom of ~100 μm after shrinkage.

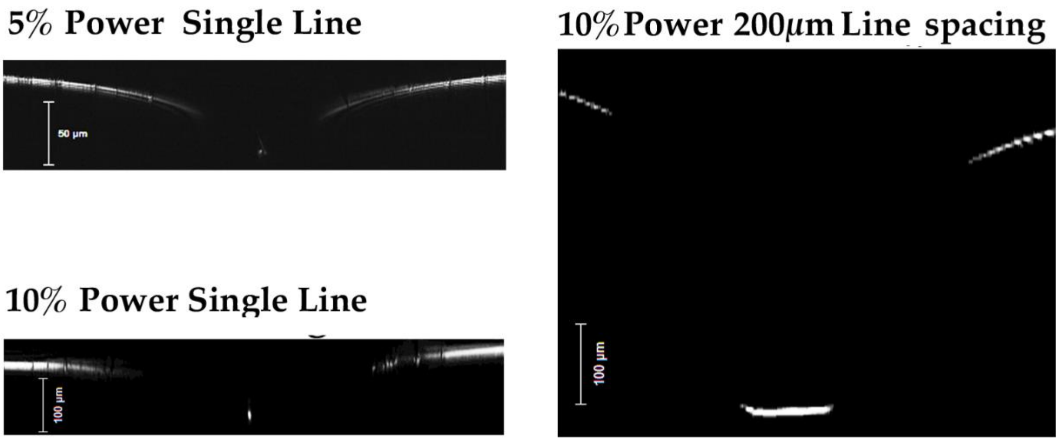

Figure C2. Effect of single vs. double line in microfluidic channel optimization. Single line design vs. double line design. Confocal microscope z-stack images of channels produced at different laser power and single line vs. double line spaced by 200 μm (channel width at the bottom of ~100 μm after shrinkage). The use of a double line design gives the channels a uniform flat bottom surface.

Appendix D: Poisson Statistical Analyses for Target Copy Number Quantification

The Poisson's probability of a droplet with zero target molecules needs to be calculated by the formula $Pr(0) = e^{-C}$, where C is the concentration of each target sample. To this end, we used the quantification of the original target sample via gel electrophoresis (see **Figure A1**).

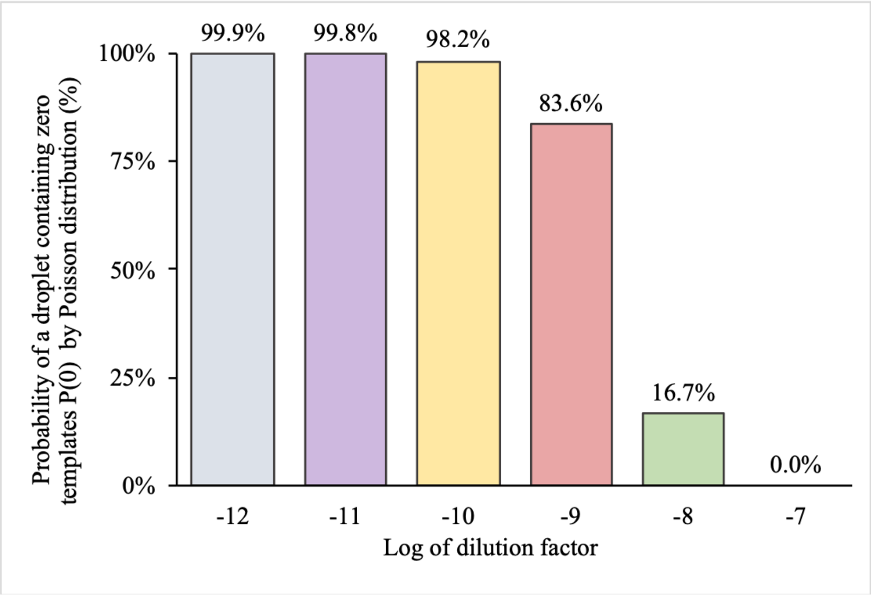

Figure D1. Poisson partitioning statistics. Probability of a droplet containing zero target molecules for each template dilution by the Poisson statistics. (●) Represents the 10^{-12} dilution, (●) Represents the 10^{-11} dilution (●) Represents the 10^{-10} dilution, (●) Represents the 10^{-9} dilution and (●) Represents the 10^{-8} dilution.
